# Towards a Controllable and Reversible Privacy Protection System for Facial Images through Enhanced Multi-Factor Modifier Networks

**DOI:** 10.3390/e25020272

**Published:** 2023-02-01

**Authors:** Yi-Lun Pan, Jun-Cheng Chen, Ja-Ling Wu

**Affiliations:** 1 Department of Computer Science and Information Engineering, National Taiwan University, Taipei 116, Taiwan; 2 National Center for High-Performance Computing, Hsinchu 300, Taiwan; 3 Research Center for Information Technology Innovation, Academia Sinica, Taipei 115, Taiwan; 4 Graduate Institute of Networking and Multimedia, National Taiwan University, Taipei 106, Taiwan

**Keywords:** anonymize (de-identification), de-anonymize (re-identification), generative adversarial networks (GAN), mutual information

## Abstract

Privacy protection data processing has been critical in recent years when pervasively equipped mobile devices could easily capture high-resolution personal images and videos that may disclose personal information. We propose a new controllable and reversible privacy protection system to address the concern in this work. The proposed scheme can automatically and stably anonymize and de-anonymize face images with one neural network and provide strong security protection with multi-factor identification solutions. Furthermore, users can include other attributes as identification factors, such as passwords and specific facial attributes. Our solution lies in a modified conditional-GAN-based training framework, the Multi-factor Modifier (MfM), to simultaneously accomplish the function of multi-factor facial anonymization and de-anonymization. It can successfully anonymize face images while generating realistic faces satisfying the conditions specified by the multi-factor features, such as gender, hair colors, and facial appearance. Furthermore, MfM can also de-anonymize de-identified faces to their corresponding original ones. One crucial part of our work is design of physically meaningful information-theory-based loss functions, which include mutual information between authentic and de-identification images and mutual information between original and re-identification images. Moreover, extensive experiments and analyses show that, with the correct multi-factor feature information, the MfM can effectively achieve nearly perfect reconstruction and generate high-fidelity and diverse anonymized faces to defend attacks from hackers better than other methods with compatible functionalities. Finally, we justify the advantages of this work through perceptual quality comparison experiments. Our experiments show that the resulting LPIPS (with a value of 0.35), FID (with a value of 28), and SSIM (with a value of 0.95) of MfM demonstrate significantly better de-identification effects than state-of-the-art works. Additionally, the MfM we designed can achieve re-identification, which improves real-world practicability.

## 1. Introduction

Since people love to use photos to record many activities and highlight personal characteristics, such as social media profiles, blog posts, vlogs, and Internet behavior, with the help of the above-mentioned personal data, face recognition can easily identify individuals and unearth personal information and then associate them with targeting individuals. Despite the accuracy rate of face recognition being lower than other competing biometric technologies, such as iris and fingerprints, due to its non-contact property, the public has widely adopted face-image-based recognition technology. However, with development of personal media and improvements in face recognition technology, a concerning situation has occurred: any random stranger can secretly collect and organize a unique identity, then associate it with specific personal information of any individual captured by the face recognition system, and then misuse or illegally sell the personal data. In 2018, the updated version of the General Data Protection Regulation (GDPR) in Europe announced stricter protection of personal data to reduce the risk of misuse of personal information, which regulates collection, storage, and use of any personally identifiable (PII) data, such as facial images, which must be carefully protected. Due to the maturity of facial recognition technology, more recently, facial photos have become the most widely used biometric feature for verifying identity (ID). However, most intelligent services driven by face recognition, such as face identity (faceID), require processing these sensitive data. There are two to-be-solved tasks related to this challenging issue. The first is to anonymize any identifiable information to protect them from abuse by malicious people, and the second is to recover them when needed unambiguously. These conflicting requirements pose an exciting trade-off between privacy and utility. Thus, to meet the goal, the design should contain two main components: anonymity (or de-identification, shortened as de-ID) and de-anonymity (or re-identification, abbreviated as re-ID).

Problems faced by de-ID and re-ID can further be addressed as follows. It is very difficult to generate high-fidelity anonymized pictures for performing de-ID without altering the original data distribution. To anonymize face images more effectively, we require a controllable and reversible system to generate a surrogate image and replace the original face, which should be a realistic and natural-looking face with the background intact while satisfying some specified conditions. In other words, the system has two preferable properties in the de-ID scenario. First, the difference between the original image and the anonymous face image should be maximized and the conversion process should be seamless and simultaneous. Second, the system should robustly provide strong security protection even when other desired features are specified forcedly. For example, users can select their hair colors to be intact for the anonymized faces as one of the preset system parameters (i.e., as part of the multi-factor features). We mainly categorize the facial attributes used in the multi-factor feature vector into two types: one is more identity-related, such as gender, age, and facial expression, and the other is more style-related, such as hair color and skin color. We denote the first kind of features identity features and the second style features.

In contrast, for completing re-ID, we also require a robust system to restore the original facial images from the de-identified ones with multi-attribute feature information (such as facial attributes and user-selected passwords). Thus, handling these specific features smoothly and successfully poses several challenging issues to the system designer. This write-up intends to address the above issues and design a reversible privacy protection system for facial images with a single neural network capable of handling a user-specified combination of features, such as passwords and facial-appearance-related attribute vectors. We name this network Multi-factor Modifier (MfM). Once training of MfM is complete, our system can fulfill the requirements of anonymization and de-anonymization at the same time. The proposed MfM can efficiently deal with correct multi-factor combinations and reconstruct near-original images. On the contrary, when receiving wrong multi-factor combinations, MfM will communicate with the facial de-ID generator to generate photo-realistic and diverse anonymized face images to prevent malicious attacks.

**Figure 1:**
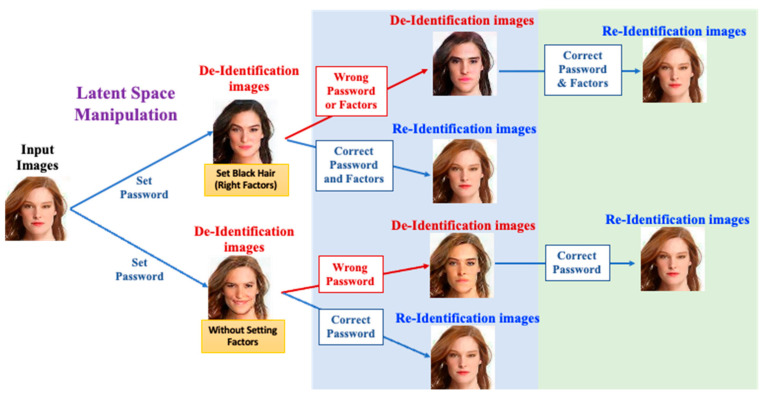
The Multi-factor Modifier for de-ID and re-ID scenario.

As shown in Figure 1, the lower portion shows the case of MfM receiving the passwords only (i.e., the single-factor case) from users to generate anonymized faces. When MfM activates the de-ID procedures, users need to input the correct passwords. With the help of a well-designed password scheme, MfM generates a near-original image. Otherwise, MfM produces another anonymized image. The upper portion of Figure 1 shows the case of MfM receiving a specific multi-factor combination as input via latent space manipulation. For example, the first and second factors may function, respectively, as passwords and face attributes, such as a female with black hair, for generating various anonymized faces. When MfM activates the de-anonymization process, users must input the correct password and correct the multi-factor combination to obtain accurate results; MfM will successfully reconstruct a near-original image with the designed password and multi-factor register schemes. Moreover, suppose users only input the correct password while using an incorrect multi-factor combination; there is no way for our system to generate the near-original image; instead, it will generate other anonymized pictures.

In summary, the proposed MfM expands the original goals [1] and extends to the following objectives—(1) maximizing feature distance between an anonymous face and its corresponding de-anonymous counterpart when the given specific multi-factor features are incorrect; (2) analyzing physical meanings of adopted cost functions based on information theory.

To justify the functionality of the proposed system and check its system performance, we train and examine the MfM work using the following datasets: FaceScrub [2], CASIA-WebFace [3], and CelebA-HQ/CelebA [4]. In Section 5, after analyzing the experimental results, we can prove that the MfM system can accomplish a multiple-task learning objective to generate synthesis-realistic anonymous images. Moreover, the MFM system can retrieve the original face images without sacrificing privacy protection performance compared with existing advanced anonymization approaches [5]. Finally, to justify our system’s ability, we investigate the quantitative and qualitative results of MfM and compare them with those of the competing de-ID techniques.

## 2. Related Work

In recent years, breakthrough of various deep generative learning models enables better face de-ID and re-ID technologies. Therefore, this section fleetingly discusses the state-of-the-art in the fields of (1) facial de-ID and facial re-ID and (2) deep face generation.

### 2.1. Facial Anonymizaiton and Facial De-Anonymization

To complete face de-ID, we must transfer various facial attributes among users, which has motivated several exciting approaches. Initially, facial-de-ID-related research tried to suppress privacy-sensitive information about a facial image by using typical image processing operations, such as pixilation, blurring, and occlusion [6,7]. Although the privacy-sensitive information did mask successfully, this approach yielded poorly anonymized faces because one has altered the data distribution significantly [7]. However, they are not widely adopted because of non-realistic anonymized quality, especially when an alteration in data distribution is not allowed. Ref. [8] proposed the method of reconstructing faces by combining a fraction of the eigenfaces to mask the ID information through the eigenvector. Ref. [9] proposed a similar method with watermarking and hashing techniques and with the principal component analysis (PCA) data representations. Some other researchers [10] used multi-factor models to unify pertinent data but needed an active appearance model (AAM) to provide extra landmarks information.

Afterward, k-Same-based algorithms [10,11,12] became the most commonly used approach for face anonymization [13]. In addition, the k-Same-based algorithms have implemented an effective k-Anonymity algorithm [14] to generate anonymous facial images. Newton et al. [11] proposed that the work could remove all personal information but generate anonymous images that are artifacts and blurring due to minor alignment errors. Recently, as deep neural network (DNN)-based learning has been becoming popular, Meden et al. [15] developed the k-Same-Net network to produce photo-realistic agent faces. Ref. [11] integrated generative neural network (GNN) and the k-Anonymity algorithm to generate high-quality images with confusion ability. Although the research is advanced in this field, there are still some aspects for improvement. First, using traditional PCA [11] causes computation to be inevitable. Second, training the GNN model is time-consuming. Finally, this research’s fatal weakness is executing down-sampling of the input images; this method impairs the quality of surrogate images. Pan et al.’s work [16], inspired by [15], proposed k-Same-Siamese-GAN to generate anonymous images without input data down-sampling. Meanwhile, Pan et al. also applied mixed precision learning to k-Same-Siamese-GAN. Therefore, k-Same-Siamese-GAN can generate high-resolution output images successfully and quickly. However, the drawback of k-Same-Siamese-GAN is that the appearance is artifactitious and unrealistic. Jeong et al. [17] resolved the unrealistic problem and provided a method with controllable features to achieve de-ID. Using controllable features also inspired us and enabled MfM to produce more diverse de-ID images through latent space manipulation.

As for de-anonymous, Yamac et al. [18] combined multi-level encryption with sensing devices to propose a reversible privacy-preserving mechanism. The strengths are in providing semi- and fully authorized schemes for decryption and a progressive augmentation learning strategy for unsupervised domain-adaptive person re-ID. The strengths above inspire our work to design an effective MfM to enhance the system’s robustness.

Gu et al.’s work [19] also inspired our work in system construction. The most significant difference between our MfM and Gu et al. is that we can complete de-ID and re-ID works simultaneously through “one” network. Moreover, we found the facial features’ choosability of [19], such as facial expression and hair color, is very limited; i.e., the resultant facial expression and hair color of the anonymized images are the same as those of the input images. If other face attributes could be parameterized, they could enhance our system’s security level. Of course, as expected, long-length passwords can provide a certain degree of security; however, the longer the password length, the more unstable and time-consuming the training process. As for diversity, Ref. [19] focused only on generating diversified anonymized images if incorrect passwords were input. Gu et al. did not discuss “controlling” the variety of generated images. Controlling diversity plays a crucial role in real-world face de-ID and re-ID applications. As pre-described, we design a single-network-based MfM to achieve anonymization and de-anonymization simultaneously. The structural and style facial image features are leveraged to enhance the system’s security level. Finally, we group the passwords and the above-mentioned facial attributes as hyperparameters to control the reconstructed images’ diversity.

In addition to the literature mentioned above, we also analyzed the state-of-the-art articles published in 2022. The more memorable part [20] achieves the effect of de-identification by integrating a quantized codebook and VQGAN/BERT models. Its strength includes dealing with integrating two large models. Still, its weakness is undeniable: the synthesized face will have a ghost effect and unnatural state during the quantization process, and the model stability is quite significant in the training challenge. On the contrary, the above problems are greatly improved in our proposed MfM. The method proposed by Yang et al. [21] mainly uses the attention model to retain essential features and uses adversarial example perturbation images to improve the diversity of anonymization images. Still, its method does not have a de-anonymization process, so it is rather limited in application. Next, the method proposed by Zhai et al. [22] is to edit the original image through attribute-aware to achieve the purpose of anonymization. However, its disadvantage is that it still needs to use other methods, such as AdaIN, to accomplish mean and variance normalization. On the one hand, it can improve the quality of image synthesis, but its disadvantage is that it makes training more complicated. Khorzooghi et al. [23] proposed using StyleGAN2 for de-identification and feature preservation. The advantage is that the synthetic face is very realistic but lacks restoration information, so there is almost no way to achieve re-identification. Xue et al. [24] use adversarial perturbations to increase the diversity of de-identification images in feature space; this article also inspired us to increase diversity through latent space manipulation.

The articles, as mentioned earlier, are all discussed in a learning-based manner, requiring more relevant theoretical analysis. In our work, we analyze and explain through theoretical methods, which is also an essential feature of this article.

### 2.2. The Deep Face Generation

Many researchers have recently studied pixel-level synthesis and editing of realistic human faces using GANs, such as [25,26,27,28]. In our work, GAN played an essential role in face image synthesis. A series of studies for improving face image generation quality also inspired our work, such as StarGAN [29] and domain-supervised GAN (DosGAN) [30] especially. In reflecting on DNNs’ progress, we built our MfM based on an unpaired image-to-image translation framework, DosGAN. DosGAN takes the first step towards exploring direct domain supervision [31]. Furthermore, to extend the diversity and address the defects of synthesized images, we include the function of StarGAN into MfM, adding extra attributes to its encoder module [29].

We use a residual net to realize our MfM, in which the discriminator of PatchGAN (D-PatchGAN) [32] is adopted to build the corresponding discriminator. PatchGAN helps restrict the MfM discriminator’s attention to the structure of local image patches.

Deep-learning-based models extract the features of the local area of the dataset image through convolution operation, so the change in the surrounding pixels will have a particular impact on the central pixel. Both will decrease the fineness of face restoration and reconstruction, which were used in past methods. The parameterization methods of image statistics include the Wallis filter algorithm, histogram normalization/matching/equalization algorithms, and the linear Monge–Kantorovich (MKL) linear color mapping algorithm, which are used to correct the pictures in the dataset. Among them, through the article of Finlayson et al. [33], we can better understand that color acts as an essential helpful information provider, especially in related applications such as tracking, object detection, and image segmentation. Its purpose is to achieve illumination invariance through histogram equalization. To understand Finlayson et al.’s work, consider using an illuminant parameter to record the RGB image through a single channel. Although the above traditional algorithms have achieved significant results, their implementation is based on the underlying low-frequency features of the image. Therefore, when the image coverage is comprehensive and the color and texture are complex, the color consistency results are more prone to color cast.

On the issue of facial alignment, and in the following research by Cao et al. [34], it is also mentioned that the more popular and successful methods are active shape model (ASM) and principal component analysis (PCA) within the active appearance model (AAM); the advantage of such methods is to minimize model parameter errors in training, making the training very efficient. However, this kind of practice has critical problems with different image orientation and creating sub-optimization. Rehman et al. [35] observed this problem and proposed using PCA to reduce the dimensionality of prominent facial features to complete facial image alignment. The central concept of [35] is to design an algorithm based on PCA and assign assignment rules inferred through the eigenvectors utilizing a two-stage approach to automate face alignment. The first stage is to segment meaningful regions according to the threshold. The second stage is to process the non-zero pixels in the binary image through PCA to determine the object rotation around the average value of the target pixels. We used MTCNN [36] and affine matrix in this study to address the above problems of facial alignment.

Therefore, this work directly uses the PatchGAN discriminator to solve the problem of high-frequency information loss so that, after being reconstructed through the MfM model, the face images taken from different light sources and angles reduce loss of useful low-frequency features in color, which better minimize the color distribution difference between the source image and the target image. Visually, the discriminator served by PathGAN can make a face synthesized by the MfM model have similar color constancy and reduce the state of color cast.

We then add several loss functions to DosGAN for constructing our MfM to conduct de-ID and re-ID processes. Meanwhile, inspired by [37], latent space-based modeling is taken for building our MfM to add the system’s diversity.

## 3. The Proposed Approach

We aim to develop a single MfM neural network to achieve a series of anonymizing and de-anonymizing processes simultaneously. With the help of latent space manipulation, users specify the password and multi-factor attributes. Then, the MfM system can generate anonymized faces when anonymization is the target. When performing de-anonymization, the MfM system receives the correct password and correct multi-factor attributes from users, then reconstructs a de-anonymized image. However, if the MfM system receives incorrect or multi-factor attributes, it will generate another agent facial image associated with a distinct identity from the original. Moreover, MfM will generate various anonymized faces without repetition in this situation. Further, the related prior knowledge can be learned from [1].

**Figure 2:**
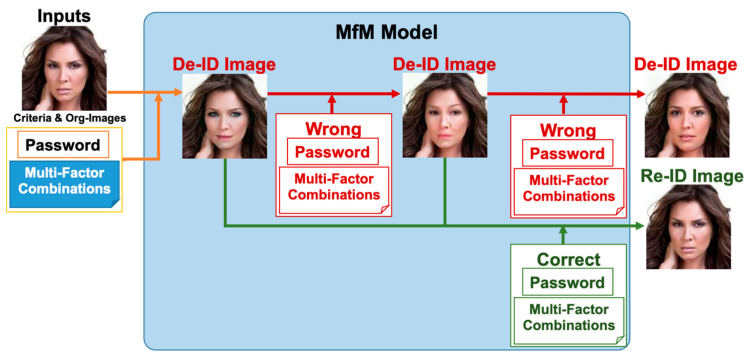
The functional descriptions of MfM model.

Figure 2 sketches the functional descriptions of MfM model. Recall that, in this work, we conduct the anonymization and de-anonymization procedures with the help of one single neural network. When activating de-ID, users need to take the hyperparameters and the original image as inputs to MfM; it generates anonymization images accordingly. When activating re-ID, users need to input correct hyperparameters to MfM again; then, it will create a re-ID face image that is near-original. Otherwise, if the received password or multi-factor combination is incorrect, MfM will change the anonymized identity into a different one and generate anonymized images that are photo-realistic and with no repetition.

### 3.1. Neural Network Architecture of Multi-Factor Modifier

**Figure 3:**
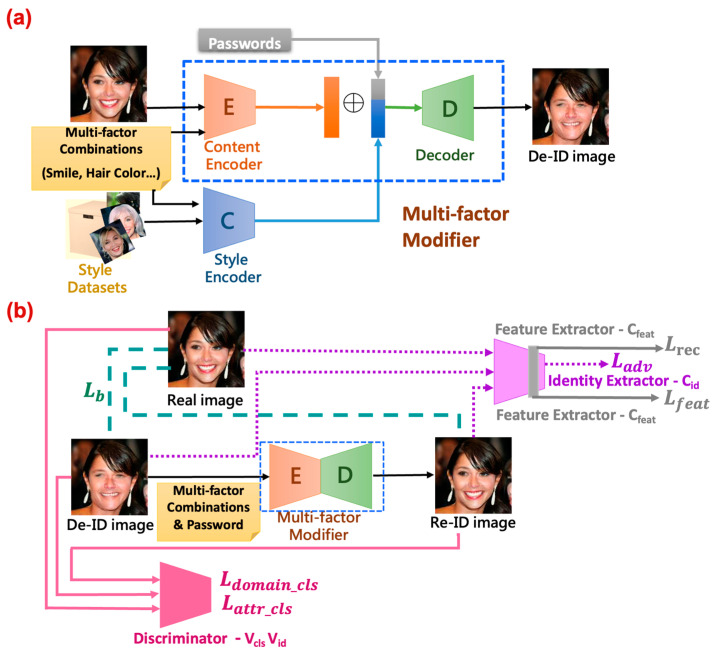
(**a**) The anonymous processes in MfM model. (**b**) The de-anonymous processes in MfM model.

Figure 3 details the neural network architecture of the MfM model for both anonymization and de-anonymization processes. As the feature shows, MfM incorporates two encoders and one decoder. One of the two encoders is the style encoder, which is the pre-trained face recognition network. The other is the content encoder, which extracts the facial-content-related features. The face IDs and face images are the inputs of the style encoder, and then the input information is fed into the so-called classification network consisting of six convolutional and three residual layers. The content encoder outputs the facial-content-related features if the classification network completes its training process. Then, MfM proceeds its operation with its two encoders and one decoder. The style encoder communicates with MfM by inputting the obtained face images and the target multi-factor combination to it. At the same time, the content encoder behaves like a residual net, which is responsible for extracting the domain identity from the outputs of the encoder’s bottleneck layer. After leveraging the style classifier’s results, MfM transmits the combination of domain identity and domain features with an extra password and multi-factor attributes. Then, the decoder generates the desired anonymous images.

The detailed operations of the proposed work can be understood as follows. The system, functional blockwise, mainly consists of the MfM and the style classifier modules. For convenience, we call the processes executed by the style classifier the phase-1 procedure of the proposed system and the MfM the phase-2. Recall that the style classifier in charge of the classification task is realized based on the multi-task D-patchGAN. First, the learned style classifier classifies the input face images into different classes according to the associated latent spaces’ features and passwords. Its output is a 1024-dimension floating-point vector, which denotes the concatenation of the latent space’s features and the user-selected passwords. Then, we go into phase-2 to generate de-identified face images satisfying the constraints guided by the pre-selected attributes.

As shown in Figure 3, the encoder of MfM model aims to reduce the dimension of the involved feature space (e.g., converting the 128 × 128 × 3 color image to 32 × 32 × 1 patches as the units for training) and then recover the domain identity (i.e., the identity associated with the de-identified image or the corresponding latent vector). In our system, the latent vector associated with the domain identity is of dimension 256 × 32 × 32. Meanwhile, the MfM’s style encoder cascades the domain features and passwords, yielding a 1024-dimension style-related latent vector. To combine both the style-related and identity-related effects, we extend the style-related latent space by repeating it 256 times and then proceeding with element-to-element-wise additions with the identity-related latent vector. Finally, we send the added results to the MfM decoder. The main task of the MfM decoder is to recover the dimension of attributes (e.g., enlarging the 32 × 32 × 1 patch image space to the original 128 × 128 × 3 color image space). Then, MfM leverages the power of D-patchGAN to judge the correctness of the input face image classification and the user pre-selected attributes. Figure 3a describes the anonymous process; when the anonymous process is completed, the discriminator will take over to calculate the local loss (the first term of Equation (1)) and turns to execute the total loss in Equation (1), as shown in Figure 3b. Thus, our proposed method just has only trained one neural network stably. Algorithm 1 addresses the detailed execution procedures of MfM.
**Algorithm 1.** Multi-factor Modifier$\mathrm{Input}:\;a\;\mathrm{set}\;\mathrm{of}\;\mathrm{face}\;\mathrm{images}\;I\;\mathrm{with}\;\mathrm{face}\;\mathrm{domain}\;\mathrm{identity}\;FaceID_{k}$$\;\mathrm{and}\;\mathrm{face}\;\mathrm{attributes}\;A_{k}$_,_∀k ∈ Φ$.\;\mathrm{Each}\;\mathrm{target}\;\mathrm{face}\;(\mathrm{de}\text{-}\mathrm{identified}\;\mathrm{face})\;F_{p}$$\;\mathrm{has}\;\mathrm{one}\;\mathrm{password}\;P_{t}$, ∀t ∈ Φ$.\;\mathrm{Training}\;\mathrm{iteration}\;J_{k}$ **Output:** Multi-factor modifier *M* and Discriminator *V*
**for***i*$=1\;\mathrm{to}\;J_{k}$**do**   $\mathrm{Randomly}\;\mathrm{select}\;\mathrm{images}\;I_{i}\;$$\mathrm{with}\;\mathrm{face}\;\mathrm{domain}\;\mathrm{identity}\;FaceID_{i}$ and randomly $\mathrm{select}\;\mathrm{image}\;I_{t}$$\;\mathrm{with}\;\mathrm{face}\;\mathrm{domain}\;\mathrm{label}\;FaceID_{t}$$.\;\mathrm{Besides},\;FaceID_{i}$ is different $\mathrm{from}\;FaceID_{t}$, ∀i, t ∈ Φ   Update Discriminator *V* with the following objective function:   $\;L_{discriminator}\;=L_{adv}+L_{rec\_cls}$   **if** (*i* + 1) mod 5 = 0 **then**     Update Modifier *M* with the following objective function:     $L_{f}=\lambda_{b}L_{b}+\lambda_{attr\_cls}L_{attr\_cls}+\lambda_{adv}L_{adv}+\lambda_{feat}L_{feat}+\lambda_{rec}L_{\mathrm{rec}}+\;\;\;\;\;\;\;\;\;\lambda_{domain\_cls}L_{domain\_cls}$     *Where*$\;L_{f}$ *defined in Equation (12)*, *respectively*.   **end if****end for**Repeat step 1 to step 10 until convergence**return***M* and *V*

Next, let us focus on the calculations of the involved loss functions. First, the loss for keeping the facial image’s appearance: we calculate the *L*_1_ distances between the de-anonymized and the anonymized facial images with the original counterpart, where *L_b_* stands for the sum of the distances mentioned in the above paragraph. Mathematically, we have

(1) $$L_{b}=\left|\left|F_{d}-I\right|{|}_{1}+\right|\left|F_{r}-I\right|{|}_{1}$$

where *I* means the original input image, *F_r_* denotes the reconstructed re-identification images, and *F_d_* denotes the de-identification images.

The second one is the attribute classification loss, denoted as *L_attr_*__*cls*_. *L_attr_cls_* is the attribute distance. That is

(2) $$L_{attr\_cls}=E_{F_{{\hat{p}}_{1}},A_{t}}[-log\;V_{cls}(A_{t}|F_{{\hat{p}}_{1}})]+E_{F_{r},A_{i}}[-log\;V_{cls}\left(A_{i}|F_{r}\right)],$$

where the cross-entropy is represented by E, and $V_{cls}(A_{t}|F_{{\hat{p}}_{1}})$ stands for conditional probability with the domain attribute labels under the given proxy image $F_{{\hat{p}}_{1}}$. *V* stands for the discriminator, which learns to classify the surrogate image $F_{{\hat{p}}_{1}}$ to its corresponding target classification domain $A_{t}$ by minimizing the objective. $E_{F_{r},A_{i}}[-log\;V_{cls}(A_{i}|F_{r})]$ denotes the domain classification loss function, where $A_{i}$ denotes the original attribute domain. Mathematically, we have

(3) $$L_{adv}=E_{F_{r},FaceID_{i}}[-logC_{id}(FaceID_{i}|F_{r})]+E_{F_{{\hat{p}}_{1}},FaceID_{t}}[-logC_{id}(FaceID_{t}|\;F_{{\hat{p}}_{1}})]\;+E_{F_{w{\hat{p}}_{1}},FaceID_{k}}[-log\;C_{id}(FaceID_{k}|\;F_{w{\hat{p}}_{1}})],$$

where $C_{id}(FaceID_{i}|F_{r})$ stands for the probability distribution over domain identity labels computed by $C_{id}$. Let $F_{r}$ denote the reconstructed image with inputting the correct password and $FaceID_{i}$ represent the ID label of the selected face image taken from the benchmark dataset. In Equation (3), we assume that the pair $\left(FaceID_{t},\;F_{{\hat{p}}_{1}}\right)$ represents the agent face generated by password p_1_ (which is denoted as $F_{{\hat{p}}_{1}}$), and $FaceID_{t}$ stands for the ID label of the generated agent face. Similarly, the pair $\left(FaceID_{k},\;F_{w{\hat{p}}_{1}}\right)$ represents the proxy face generated by giving the wrong password wp_1_ (which is denoted as $F_{w{\hat{p}}_{1}}$), and $FaceID_{k}$ is the ID label of the proxy face generated by using the wrong password $w{\hat{p}}_{1}$.

Moreover, we design loss functions for measuring the dissimilarity between images in the following situation: (i) with different passwords situation, (ii) with wrong given passwords situation, and (iii) the de-ID face and the other de-ID face with wrong password situation. Mathematically, we have

(4a) $$L_{feat}=L_{dis}\left(C_{feat}\left(F_{{\hat{p}}_{1}}\right),C_{feat}\left(F_{{\hat{p}}_{2}}\right)\right)+L_{dis}\left(C_{feat}\left(F_{{\hat{p}}_{1}}\right),C_{feat}\left(F_{w{\hat{p}}_{1}}\right)\right)\;+L_{dis}\left(C_{feat}\left(F_{{\hat{p}}_{1}}\right),C_{feat}\left(F_{w{\hat{p}}_{2}}\right)\right),$$

where

(4b) $$L_{dis}\left(C_{feat}\left(F_{{\hat{p}}_{1}}\right),C_{feat}\left(F_{{\hat{p}}_{2}}\right)\right)=max\left(0,cos\left(C_{feat}\left(F_{{\hat{p}}_{1}}\right),C_{feat}\left(F_{{\hat{p}}_{2}}\right)\right)\right).$$

In Equation (4), ‘cos (x, y)’ denotes the cosine similarity between two vectors x and y, and $C_{feat}\left(F_{{\hat{p}}_{1}}\right)$ and $C_{feat}\left(F_{{\hat{p}}_{2}}\right)$ are two transformed face images associated with two different passwords p_1_ and p_2_, respectively.

Mathematically, the corresponding reconstruction loss is shown as following

(5) $$L_{rec}=||C_{feat}\left(I\right)-C_{feat}\left(F_{r}\right)|{|}_{1}$$

We use the *L*_1_ distance to calculate the domain classification loss, which is $L_{domain\_cls}$. It is obtained through the discriminator *V_id_* and obtained through the *C_id_* (also called identity extractor). Thus, we have

(6) $$L_{domain\_cls}=||V_{id}\left(F_{{\hat{p}}_{1}}\right)-C_{id}\left(F_{{\hat{p}}_{1}}\right)|{|}_{1}$$

Finally, the total objective loss can be written as:

(7) $$L_{f}=\lambda_{b}L_{b}+\lambda_{attr\_cls}L_{attr\_cls}+\lambda_{adv}L_{adv}+\lambda_{feat}L_{feat}+\lambda_{rec}L_{\mathrm{rec}}+\lambda_{domain\_cls}L_{domain\_cls}$$

The following parameter settings are used in this work, including *λ_feat_* = 10, *λ_adv_* = 1, *and λ_b_* = *λ_attr cls_* = *λ_rec_* = *λ_domain cls_* = 20.

### 3.2. The Related Schemes—Password and Multi-Factor Register

We designed the password scheme to associate the face ID in datasets with an N-bit password (*p* ∈ (0,1)*^N^*). Our password scheme is inherited from [19], but we change the implementation using the following combination of latent codes. That is

1. $\hat{C}\left(F_{r},A_{i}\right)\equiv C\left(F_{r},A_{i}\right)⨀P_{t}$, where $C\left(F_{r},A_{i}\right)\in {\mathbb{R}}^{1024}$ denotes the outputs of the latent codes of the content encoder and ⨀ denotes the Concatenate operation.
2. $P_{t}$ is binary string and enables 2*^N^* unique passwords.

The other is the multi-factor register scheme. It is responsible for recoding identity-related face attributes and style-related (non-identity-related) face attributes.

Similar to the anonymization scenario, our system needs two more steps to activate the de-anonymization processes. The first step is using a lookup table to record the specific password and multi-factor attributes from users’ inputs. The second step is applying a neural-network-based training process to include the selected features’ effects in our system and proceed with the final reconstruction step.

## 4. Information-Theoretic-Based Analyses and Discussions of MfM

### 4.1. Physical Meanings of the Adopted Cost Functions

There are two specific cost functions to guide the learning of MfM, which, respectively, take our system’s visual acceptability and recovery radiality into consideration. The following minimax game is the visual acceptability regularization function. That is

(8) $$min_{M}max_{D}V_{I}\left(D,M\right)=V\left(D,M\right)-\lambda_{1}I\left(c;M\left(P,\;c\right)\right),$$

where *I*(x, y) is the mutual information to measure the relationship of two random variables x and y, *D* is the discriminator, *M* denotes the MfM model, $\lambda_{1}$ is a hyperparameter, c stands for the latent codes of the original face image, and *P* $=\{p_{1:\mathrm{password}},\;p_{2:\mathrm{multi}-\mathrm{factor}\;\mathrm{combinations}}\}\;(≜\{p_{1},\;p_{2}\}$ for short) is the set of embedding information that consists of the given password $p_{1}$ and the given multi-factor combinations $p_{2}$. Then, we can form the affinity between the original face image c and the anonymous image $Z_{\mathrm{nid}}$ into $I\left(c;\;Z_{\mathrm{nid}}\right)=I\left(c;\;M\left(P,\;c\right)\right)$ after processing through the MfM model.

We regularize the above minimax game by maximizing the mutual information (MI) between our model representations of the original face image and the de-ID image to bound the tolerable visual difference between them. We treat *P* as a random variable in the following discussions. From the fundamental relations between MI and entropy, we have

(9) $$I\left(X;\;Y\right)=H\left(X\right)-H\left(X|Y\right)=H\left(Y\right)-H\left(Y|X\right).$$

We expressed $I\left(c;Z_{\mathrm{nid}}\right)=I\left(c;M\left(P,c\right)\right)$ as the distribution distance, which is measured between the original facial images and the anonymous images. Due to a deterministic and invertible encoding function, *M*(.), relates c and $Z_{\mathrm{nid}}$, and the maximal value of $I\left(c;Z_{\mathrm{nid}}\right)$ can be derived. That means the cost function can be constrained because the visual difference between c and $Z_{\mathrm{nid}}$ is controlled within an acceptable range. This property is an essential requirement for privacy protection. The above interpretation also represents latent code (c) containing meaningful information, and the latent code will not be lost too much during the MfM model training. According to Equation (9), $I\;\left(c;Z_{\mathrm{nid}}\right)$ is denoted:

(10) $$I\left(c;Z_{\mathrm{nid}}\right)=I\left(c;M\left(P,c\right)\right)=H\left(c\right)-H\left(c|M\left(P,c\right)\right).$$

*M*(.) is complicated to find the maximal value of Equation (10) because of needing to capture the posterior $p\left(c|M\left(P,c\right)\right).$ information; even *M*(.) is deterministic and invertible. With the help of using a variational approximation as follows, we can solve the computation difficulty of encoder mutual information.

The distribution of data x is $p\left(x\right)$, and the following question is how to bound $H\left(c|M\left(P,c\right)\right)$ suitably. We use the positive property of the Kullback–Leibler (KL) divergence:

(11) $$\sum_{c}p(c|M\left(P,c\right))log\;p(c|M\left(P,c\right))-p(c|M\left(P,c\right))log\;q(c|M\left(P,c\right))\geq 0,$$

where the arbitrary obtainable variational distribution is expressed as $q(c|M\left(P,c\right))$. Thus,

(12) $$I\left(c;M\left(P,c\right)\right)=H\left(c\right)-H\left(c|M\left(P,c\right)\right)\geq H\left(c\right)+{\langle logq\left(c|M\left(P,c\right)\right)\rangle }_{p\left(c,M\left(P,c\right)\right)}≜\tilde{I}\left(c;M\left(P,c\right)\right),$$

where $H\left(c\right)=-{\langle logp\left(c\right)\rangle }_{p\left(c\right)}$, $H\left(c|M\left(P,c\right)\right)=-{\langle logp\left(c|M\left(P,c\right)\right)\rangle }_{p\left(c,M\left(P,c\right)\right)}$, and $\tilde{I}\left(c;M\left(P,c\right)\right)$ is an approximation of $I\left(c;M\left(P,c\right)\right)$ based on $q(c|M\left(P,c\right))$. The relation indicated in Equation (12) is equivalent to describing a moment-matching approximation of $p\left(c|M\left(P,c\right)\right)$ by $q(c|M\left(P,c\right))$.

$M\left(P,c\right)$ is as an information channel. The inputs of $M\left(P,c\right)$ are P and c. The output of $M\left(P,c\right)$ *is* $Z_{\mathrm{nid}}$. The conditional probability of constructing $Z_{\mathrm{nid}}$ under given *c* can be expressed as:

(13) $$logp\left(Z_{\mathrm{nid}}|c\right)=log\int_{M\left(P,c\right)}^{}p\left(Z_{\mathrm{nid}}|M\left(P,c\right)\right)p(M\left(P,c\right)|c)\;\geq \;{\langle log\;p(Z_{\mathrm{nid}}|M\left(P,c\right))\rangle }_{p(M\left(P,c\right)|c)}.$$

After averaging Equation (13) over all possible c and combining with the approximation results obtained from Equation (11), we have

(14) $$\sum_{c}p\left(c\right)logp\left(Z_{\mathrm{nid}}|c\right)\;\geq \;\sum_{c}{\langle log\;p(Z_{\mathrm{nid}}|M\left(P,c\right))\rangle }_{p\left(c,M\left(P,c\right)\right)}\approx {\langle log\;q(c|M\left(P,c\right))\rangle }_{p\left(c,M\left(P,c\right)\right)}$$

By exchanging the terms on the different sides of Equation (12), it is:

(15) $$H\left(c|M\left(P,c\right)\right)\geq H\left(c\right)-\tilde{I}\left(c;M\left(P,c\right)\right)$$

When giving $M\left(P,c\right)$ measured based on $q(c|M\left(P,c\right))$, we can derive the lower bound of the latent code c’s perdition error according to Equation (15). If fixed $p\left(c\right)$, we can find the maximization of $I\left(c;M\left(P,c\right)\right)$ measured based on $q\left(c|M\left(P,c\right)\right)$ is equivalent to computing the desired lower bound. Therefore, we obtained the visual acceptability cost function, as shown in Equation (8). Similarly, we can write the recovery radiality cost function as another minimax game. That is

(16) $$min_{M}max_{D}V_{I}\left(D,\;M\right)=V\left(D,M\right)-\lambda_{2}I\left(c;M\left(Z_{nid},\;\hat{P}\right)\right),$$

where $\lambda_{2}$ is a hyperparameter and $\hat{P}$ stands for the input password and multi-factor combinations from users or hackers.

For the case $\hat{P}=P$, according to Cheng, et al. [38] and with the aid of a variational marginal approximation $r\left(M\left(Z_{nid},P\right)\right),$ which is a standard normal distribution for building the variational upper bound, we have

(17) $$I\left(c;Z_{id}\right)=I\left(c;M\left(Z_{nid},\;P\right)\right)\;\;\;\;\;\;\;\;\;\;\;\;\;\;\;=E_{p\left(c,\;M\left(Z_{nid},\;P\right)\right)}\left[log\frac{p(M\left(Z_{nid},P\right)|c)}{p\left(M\left(Z_{nid},P\right)\right)}\right]\;=E_{p\left(c,\;M\left(Z_{nid},\;P\right)\right)}\left[log\frac{p\left(M\left(Z_{nid},P\right)|c\right)}{r\left(M\left(Z_{nid},P\right)\right)}\right]-KL(p\left(M\left(Z_{nid},P\right)\right)||r\left(M\left(Z_{nid},P\right)\right))\;\leq E_{p\left(c,\;M\left(Z_{nid},\;P\right)\right)}\left[log\frac{p\left(M\left(Z_{nid},P\right)|c\right)}{r\left(M\left(Z_{nid},P\right)\right)}\right]=KL(p(M\left(Z_{nid},P\right)|c)||r\left(M\left(Z_{nid},P\right)\right)).$$

Now, assume the designed regulation function constrains the $KL(p\left(M\left(Z_{nid},P\right)\right)||r\left(M\left(Z_{nid},P\right))\right)$ to become very small, which means the learning process will force $r\left(M\left(Z_{nid},P\right)\right)$ to become a well-density approximation of $p\left(M\left(Z_{nid},P\right)\right)$. In other words, such a regulation function will help complete the re-ID process very positively. Similar arguments hold for the case of $\hat{P}\neq P$; we now want to bound $H\left(c|M\left(Z_{nid},\hat{P}\;\right)\right).$ With the definition of mutual information and the positive property of the Kullback–Leibler divergence, we have:

(18) $$I\left(c;M\left(Z_{nid},\hat{P}\;\right)\right)=H\left(c\right)-H\left(c|M\left(Z_{nid},\;\hat{P}\right)\right)\geq H\left(c\right)+{\langle logq\left(c|M\left(Z_{nid},\;\hat{P}\right)\right)\rangle }_{p\left(c,M\left(Z_{nid},\hat{P}\right)\right)}≜\tilde{I}\left(c;M\left(Z_{nid},\;\hat{P}\right)\right),$$

where $q\left(c|M\left(Z_{nid},\;\hat{P}\right)\right)$ is another obtainable variational distribution. Once again, our well-designed regulation function will lead the learned $q\left(c|M\left(Z_{nid},\;\hat{P}\right)\right)$ approximating to $p\left(c|M\left(Z_{nid},\;\hat{P}\right)\right)$ closely, which means the relation indicated in Equation (18) is equivalent to a moment matching approximation of $p\left(c|M\left(Z_{nid},\;\hat{P}\right)\right)$ by $q\left(c|M\left(Z_{nid},\;\hat{P}\right)\right).$ Through the discussions presented above, we expect to give readers a better understanding of the cost functions we used in the proposed MfM scheme.

### 4.2. Latent Space Manipulation for MfM

To demonstrate the ability of the claimed control diversity, we proceed with the analysis in latent space. As Figure 3 illustrates, the style encoder generates the domain-feature-specific latent space (the green region in Figure 3a). In contrast, the content encoder creates the domain-identity-specific latent space (the blue region in Figure 3a). That is, we represent the identity-related and style-related features of a given face image by using their corresponding latent codes in the domain-specific latent space.

**Figure 4:**
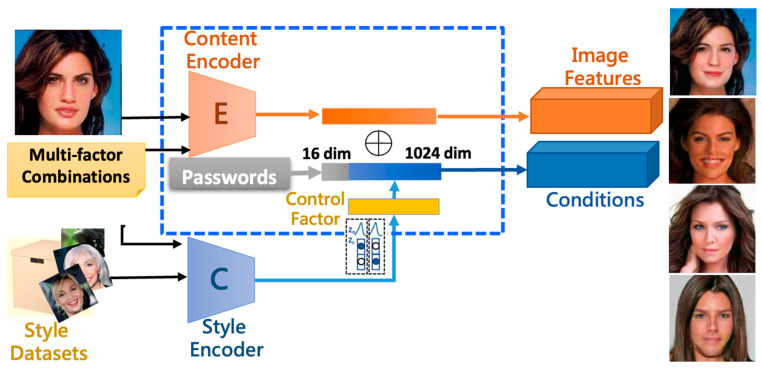
The proposed controller-added clustering network.

We include the concept of clustering in our latent space analytics. Moreover, to increase the variety of the generated agent faces, it is critical to have both clustering and interpolation abilities. We use the MfM model to achieve the goals for clustering the latent space. Since we already knew that the style encoder’s outputs do affect the identity of the de-ID images, we, therefore, add a controlling factor for changing the grouping conditions for generating the specific features. It depicts the proposed controller-added clustering network (Figure 4).

We apply the PCA to the benchmarking dataset (with 6000 identities originally) to perform feature extraction, dimension reduction, and effective cluster grouping. With the benefit of PCA, we can guide the direction of attributes distribution and avoid attributes entanglement. We use t-SNE (t-distributed stochastic neighbor embedding) to implement the PCA efficiently. Algorithms such as t-SNE only care about pairwise distances between points. The t-SNE tries to position the points on a plane such that the pairwise distances between them would minimize a particular criterion. Thus, to see the effectiveness of clustering, we designed the following three testing cases with different attribute (or equivalently the labeled feature) numbers for further analyses:

1. We select only four style-related features to group the dataset for a chosen identity.
2. We increase the number of features from four to twenty, in which fifteen style-related (i.e., homogeneous) and five identity-related (i.e., inhomogeneous) attributes associate with five selected identities.
3. We mixed the style-related and identity-related features for grouping the dataset.

**Figure 5:**
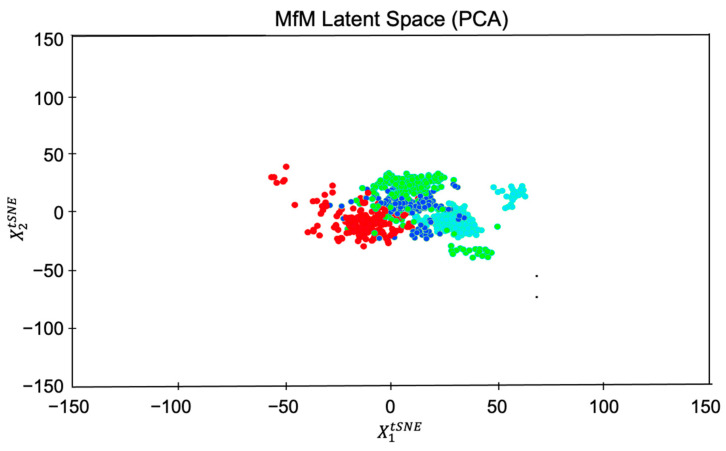
Clustering based on four chosen homogeneous features (the hair color attribute in this example).

**Figure 6:**
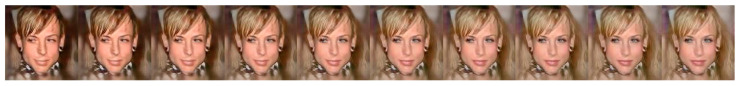
Perceptual effects of the clustering evolution associated with Figure 5 (from left to right, the hair colors changed from black to blond).

Case 1. We obtained four clusters according to the four selected homogeneous features (with labels for training)—black hair, brown hair, gray hair, and blond hair, as illustrated in Figure 5. It displays the steady state of the whole group evolution. To visualize the effect of PCA-based clustering, we also snapshot the hair-color-changing evolution by showing the photos associated with each of the four groups’ cluster centers in Figure 6. The generated agent faces’ hair colors gradually change from black to blond from left to right.

**Figure 7:**
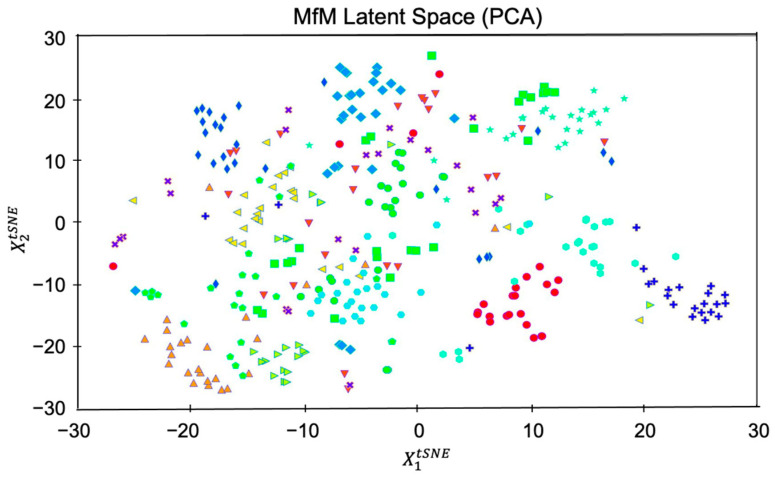
Clustering based on twenty chosen inhomogeneous features, as listed in Table 1.

**Figure 8:**
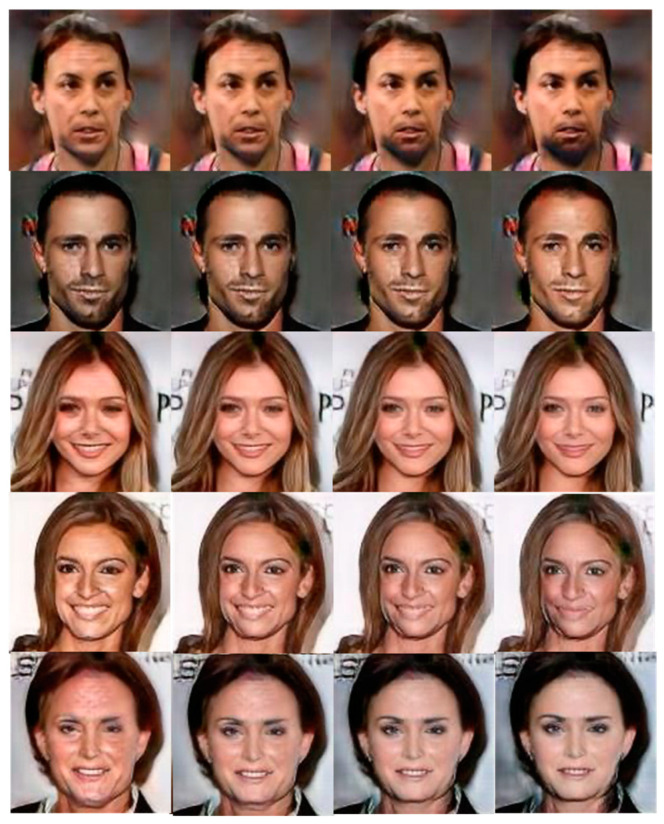
Perceptual effects of the clustering evolution associated with testing case 2.

Case 2. We now use twenty attributes, including identity-related and style-related attributes, to group datasets. In other words, we perform PCA-based dimension reduction on the tested dataset and achieve twenty clusters via the twenty labeled features listed in Table 1. Similar to Figure 5, we depict the grouping with twenty mixed attributes in Figure 7. Since both identity- and style-related attributes are considered, the figure displays the steady-state of the whole group evolution. Moreover, we also illustrate the evolution of agent faces’ changing via snapshotting the twenty group-centric face images. As shown in Figure 8, the beards on faces of the same identity in the first row grow thicker from left to right, and those in the second row are getting thinner. In the third row, the mouths change from open to closed, and those in the fourth row change from happier to neutral. Most interestingly, the faces in the fifth row switch gender gradually.

**Figure 9:**
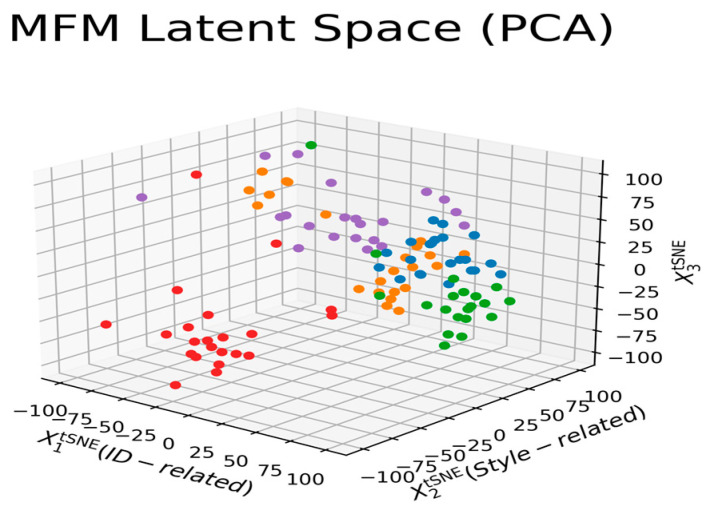
Clustering based on the correlation of both style-related and identity-related attributes.

To better analyze and view various attributes’ effects on the clustering process, we redraw Figure 7 according to the correlation of different labeled features in Figure 9. ‘X1’ and ‘X2’ axes of Figure 9, respectively, denote the distributions of pairwise distances between points (in the PCA space) of different identity-related and style-related attributes. At the same time, the ‘X3’ axis represents the feature’s correlation strength. Therefore, the five clearly grouped clusters in the figure indicate there are five identities with various degree style-related characteristics. Let us explore the results of Figure 9 in a bit more detail. The colors associated with the five clusters imply that each identity has a dominant representative hair color, that is, blue, green, orange, purple, and red for identity 1, 2, 3, 4, and 5, respectively. Figure 9 also reflects that each cluster has an apparent trait that affects the appearance of the generated agent face. Moreover, the tighter the clustering density, the stronger the effect is. Thus, the evolution processes converge to five identities with different facial appearances, which matches the results demonstrated in Figure 8. Let us take the blue- and green-colored groups as examples; since the X1-axis’s standard deviation of these two groups is relatively less significant than that of the other groups, there are no gender changes, as shown in the top two rows of Figure 8.

**Figure 10:**
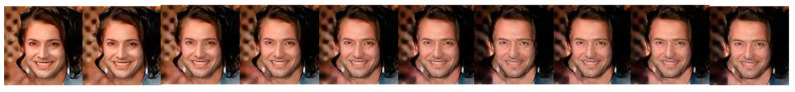
Using multiple attributes to generate agent faces: gender: from female to male; age: from young to old; mustache: from no mustache to mustache.

To further illustrate the effects of various attributes on the generated agent faces, we also snapshot the results of testing case 3 in Figure 10, where many style-related and identity-related features are mixed for a selected identity. For example, from left to right, as shown in Figure 10, the agent faces change from old to young, genders from male to female, and hair colors from gray to black. After analyzing the above three testing cases, we find that the degree of effect on the final agent’s appearance of an attribute is inversely proportional to the absolute value of its variance. This observation implies that developing effective and efficient face-image editing mechanisms in the latent space by adequately combining the selected attributes with robust machine learning schemes is worthy of completing.

We try to find the bottleneck features and fit them into image manipulations in the latent space by taking MfM as the pre-trained model. The controller-added clustering network encodes the original image into intermediate features. Next, we execute the element-wised addition of the features by using the guidance vectors, which are generated based on the given operating (manipulation) conditions. At last, the modified bottleneck features can influence the decoder to generate the manipulated images. The following experiments prove the proposed scheme is still workable when we add some constraints to bound the latent space. The experimental results also justify that the designed regulations directly manipulate images in the latent space.

**Figure 11:**
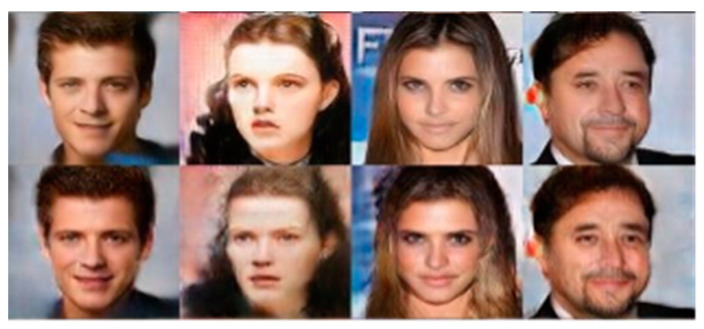
(**Top**) Based on the MfM model manipulating images snapshots and (**bottom**) top-row images after compressing and decompressing the bottleneck features’ corresponding snapshots.

**Figure 12:**
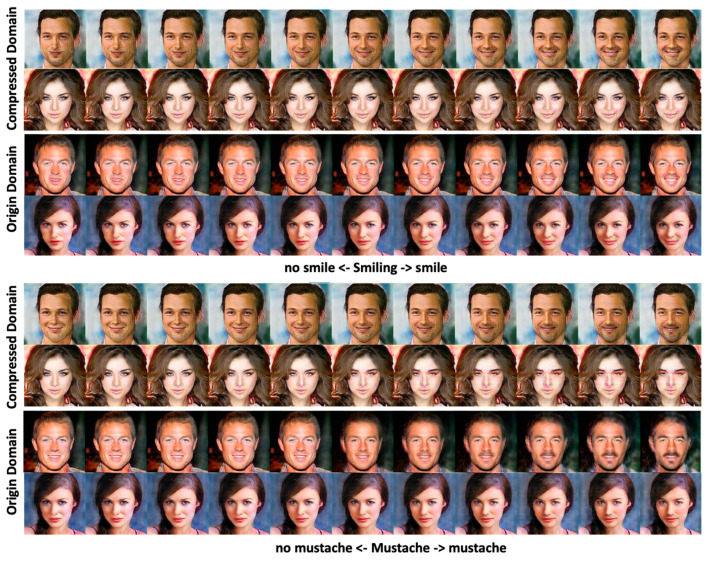
The shown varying degrees of smiling (**top two rows**) and the mustache (the **bottom two rows**) attributes changing of the MfM model-based manipulated results.

**Figure 13:**
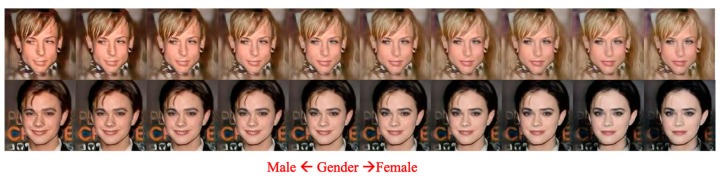
The shown varying degrees of the gender attribute changing of the MfM model-based Manipulated results.

It takes the MfM as an encoder and performs well in facial-related images. The reason is that it is more likely to preserve high-frequency facial information when using the MfM model as the encoder, even if we perform the image’s compression to a certain level. From the snapshots presented in Figure 11, Figure 12 and Figure 13, there is not much difference between the manipulated images and their original counterparts since they are rebuilt by decompressing the shifting guidance-added and MfM-generated bottleneck features.

## 5. Experimental Results

To demonstrate the proposed approach’s effectiveness, we conduct quantitative and qualitative experiments and compare them with other existing works.

### 5.1. The Training Datasets, Testing Datasets, and Evaluation Metrics

We used the face verification toolkits [39,40] to measure the distance between de-ID images and the original face images. Meanwhile, we also used Facenet, insightface, insightface-ir50_Asia, and insightface-ir50_ms1m networks for testing. On the other hand, we also applied the face recognition toolkit [41] to compare the similarity of re-ID images to the original face images.

In training, we individually use 85% of the FaceScrub, CASIA-WebFace, and CelebA-HQ/CelebA datasets. The remaining 10% of the above datasets are used for testing, and the last 5% for validation. FaceScrub is one of the most extensive public facial datasets, which contains 530 male and female celebrities; each person has 200 images, and the total number is 106,863 facial images. Since each person has about 200 pictures, the MfM model can learn face attributes with more diversity and more easily apply them to different datasets. Further, the CASIA-WebFace dataset has more than 453,453 facial images of 10,575 people, and the CelebA-HQ/CelebA has more than 30,000 facial images of 10,177 people.

After collecting the required datasets, next, we need to discuss the pre-processing of facial images in the datasets. Especially with the MfM model, we designed deals with new data, such as video screenshots or CASIA-WebFace; alignment is also a critical key point that affects the quality of the training model, and face alignment is necessary pre-processing in many face applications. Its purpose is to reduce loss of accuracy caused by the input image’s rotation, translation, and scale. Furthermore, in practice, the better the face alignment is, the more direct and intuitive the impact on the quality of image synthesis performed by our MfM model. Since the data are screenshots from videos or the CASIA-WebFace, there will be images with different angles or occluded facial features in datasets. We can use PCA to reduce the datasets’ dimensionality, project it to 2D coordinates, and perform the grouping action on similar coordinates to filter out faces from different angles or occluded faces. Taking facial orientation as an example, random facial orientation can be filtered into standard facial orientation. Facial orientation alignment can be completed through practice of this part.

Considering the above discussion, go through the following steps to process the normalization of the facial images:

Step 1. Dealing with Bounding Box: This part aims to extract the face.

Step 2. Creating Landmarks: We use the MTCNN [36] network to locate facial features and generate landmarks.

Step3. Using Affine Matrix to normalize the facial images: it aims to rotate, translate, and scale in on facial images and then crop the raw facial images into facial images with the size of 256 × 256.

After completing the above steps, the designed MfM model can start training.

In the evaluation part, we used perceptual-based image quality metrics: learned perceptual image patch similarity (LPIPS), Frechét inception distance (FID), and structural similarity index measure (SSIM). LPIPS is used to assess image patches’ distance. Higher LPIPS means further/more different and vice versa. FID is a measure of similarity between two datasets of images. Meanwhile, it was shown to correlate well with human judgment of visual quality and is most often used to evaluate quality of samples of GANs. On the other hand, we also use SSIM as a performance index for measuring similarity of two images.

### 5.2. Quantitative Results and Discussion of the Proposed Multi-Factor Modifier

At first, we referred to the experiments conducted on StarGAN v2 [42] and implemented the works of Gu et al. [19], Maximov et al. [43], and Cao et al. [44] for broadening our comparison targets. Then, we compared the outcomes of [16,19,43] and [44] with that of our work.

According to Table 2, the proposed MfM system performs better than state-of-the-art works. In addition, because of the higher value in LPIPS, it means a higher diversity degree of generated images. This is also the essential property of anonymization. Furthermore, our system’s super performance in FID is the most crucial observation. In general, the lower the FID value the better because FID indicates the distance between two distributions of the real and the generated images. Further, we also compare SSIM and the results show the de-ID image quality of the MfM model is relatively good.

Following, let us compare the proposed work with the anonymization performance of other de-ID-related works, including CIAGAN, k-Same-Net, and k-Same-Siamese-GAN.

**Figure 14:**
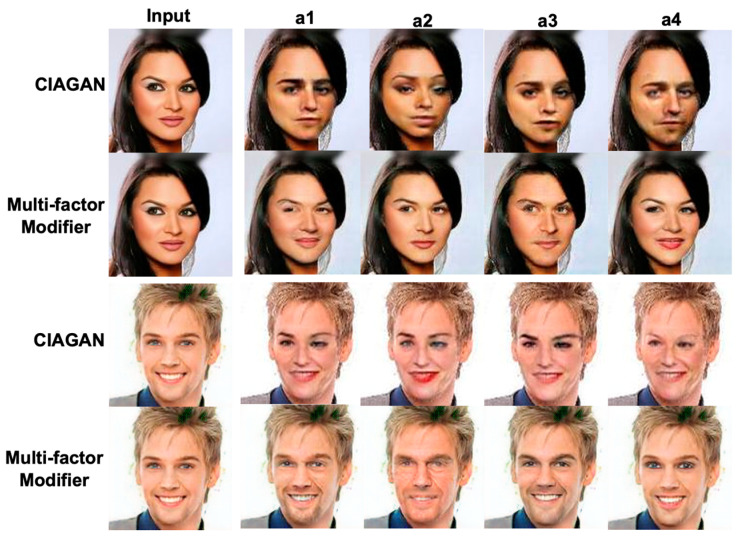
The visual comparison of polluted situations in the synthesized images between competing systems, including CIAGAN and MfM.

We focus on comparison between CIAGAN and MfM. In Figure 14, the odd-numbered rows are the surrogate faces generated by CIAGAN, and the even-numbered rows are the surrogate faces generated by MfM. Through experiments, it can be found that the surrogate faces generated by CIAGAN have abnormal states, among which eye angle and lips are the most eccentric and unrealistic. For example, analyzing the synthesized eye angles, we can find that the eye angles will be affected by the closed hair, so the synthesis of the right eye will be skewed, and these anonymous images generated by CIAGAN are very different from the original ones. Therefore, this kind of state can be confirmed on the MfM model that there is no such situation. Furthermore, in the third row of Figure 14, the lips synthesized by CIAGAN also show an extremely unnatural state, which shows a thick red lip. Similarly, we also confirmed that the surrogate faces generated by the MfM model appear more natural in the partial effect of lips.

**Figure 15:**
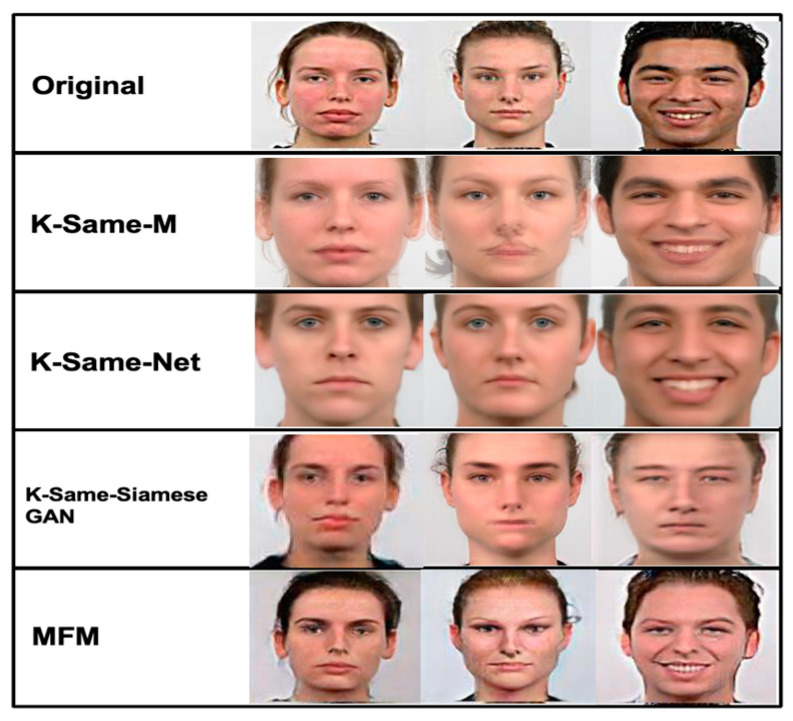
The snapshots of de-ID images generated by k-Same-M, k-Same-Net, k-Same-Siamese-GAN, and MfM.

To check the de-ID performance of the MfM model, we compare the de-ID images’ quality generated by k-Same-M, k-Same-Net, k-Same-Siamese-GAN, and MfM. In Figure 15, the row from top to bottom represents the original image and rendered k-Same-M, k-Same-Net, k-Same-Siamese-GAN, and MfM, respectively. Due to the different schemes, the information content will also be different, and the visual quality of the generated images will also be various. Among them, the surrogate face generated by the MfM model is more natural and smoother, which means that its high-frequency facial information is better preserved, and such characteristics will also assist image quality after re-ID.

As shown in Figure 15, MfM provides the best result in terms of image quality. Furthermore, its de-anonymous capability has also been implemented, which is very different from the three k-Same series works. As shown at the bottom of Figure 15, the proposed approach provides realistic and high-quality synthesis images and supports an anonymity and de-anonymity guarantee.

**Figure 16:**
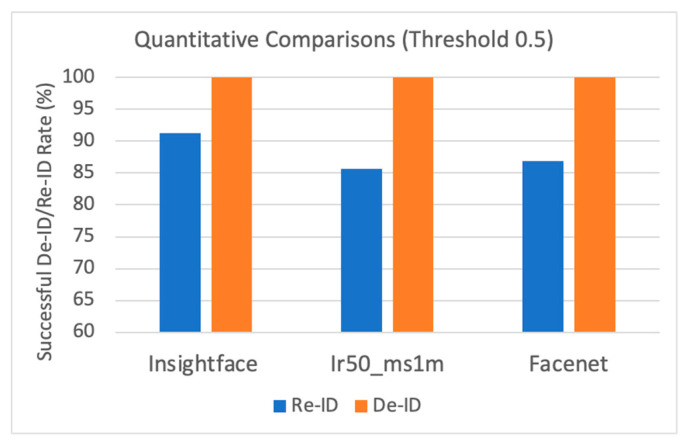
Quantitative comparisons: successful de-ID/re-ID rate.

We then compared the original images with both the anonymous images and the de- anonymous images using the face recognition methods, including insightface, insightface_ir50 ms1m, and Facenet. If the generated de-anonymous image is consistent with the original, we indicate in this case a score of 0. On the other hand, if the generated de-ID image is inconsistent with the original, we provide a score of 1. In Figure 16, we found that the three networks showed 100% on the successful de-ID images, which means that the de-ID images generated by MfM with a pre-specified similarity threshold of 0.5 were successfully identified differently from the original images. Under the de-anonymization situation, the MfM system’s outputs achieved a relatively positive gain in insightface (91.3%). That is, the proposed MfM produces the best de-ID and re-ID results when using the insightface model. We also report that the RMSE value is 0.353, and the accuracy value is 0.875.

**Figure 17:**
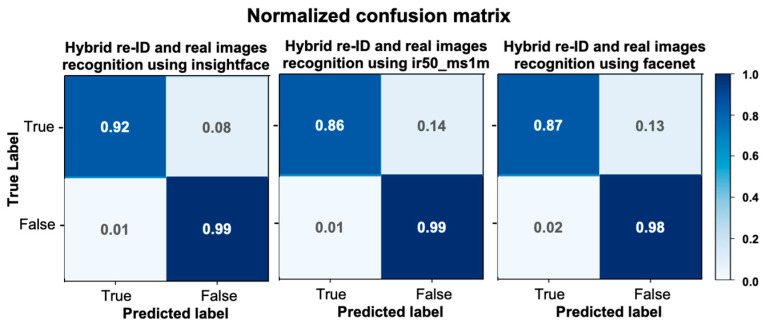
The normalized confusion matrix to evaluate MfM model for re-ID.

Last, we also added the confusion matrix to evaluate the validity of the MfM model during the de-anonymous process. In Figure 17, the normalized confusion matrix shows that the proposed method performs exceptionally well on the three networks.

### 5.3. Qualitative Results of the Proposed Multi-Factor Modifier

This sub-section presents more qualitative evaluation results of MfM concerning utility-related experiments.

**Figure 18:**
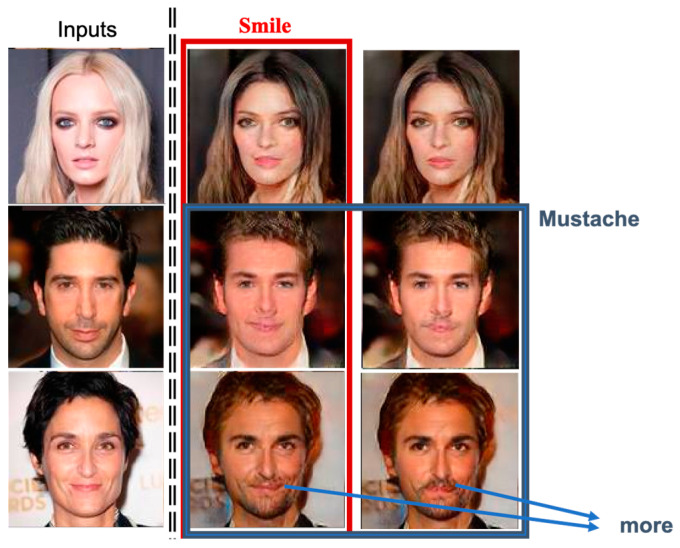
The generated agent faces using identity-related attributes: smile and mustache.

First, we take facial expressions and mustache into account. In Figure 18, the first column shows the input images, and the second column includes one identity-related facial attribute, i.e., smile facial expression; thus, the generated surrogates are with smiles. The snapshots shown in the third column reflect the effects of another identity-related facial attribute, mustache. Notice that our system also detects the attribute gender of the input image, and, since the detected gender is female, our system is reluctant to include the mustache attribute in the generated surrogate; when the chosen attributes conflict with each other, our system will generate a less unreasonable anonymized face.

**Figure 19:**
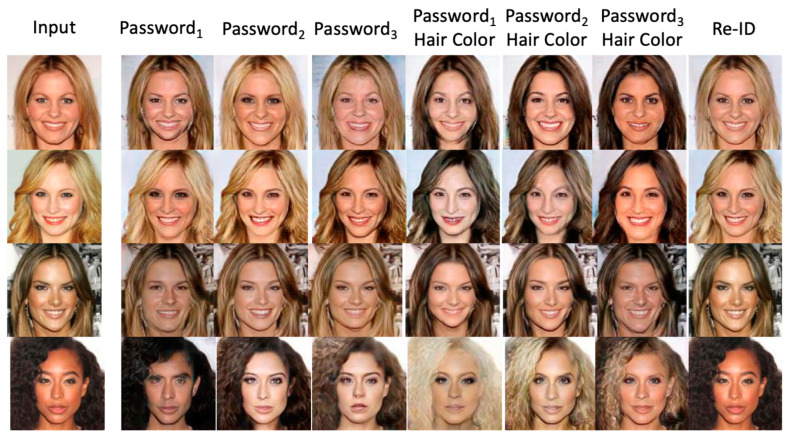
The agent faces generated using style-related face attributes, such as password and hair color.

Now, let us examine the effect of using style-related face attributes, such as password and hair color. In Figure 19, the first column shows the input images, and the second to fourth columns show the resultant surrogates corresponding to different passwords with various numbers of bits. The fifth to seventh columns show the de-ID images obtained using the multi-factor combination scheme that jointly takes password and hair color into account. Finally, the eighth column depicts the excellent picture quality of re-ID results. In other words, the results also prove that we can reconstruct good de-anonymized pictures. Furthermore, leveraging the facial recognition tool and the proposed MfM system, the distances between the anonymized images and the input images are very far, as shown in Figure 19, which means the quality of anonymized images will be excellent.

**Figure 20:**
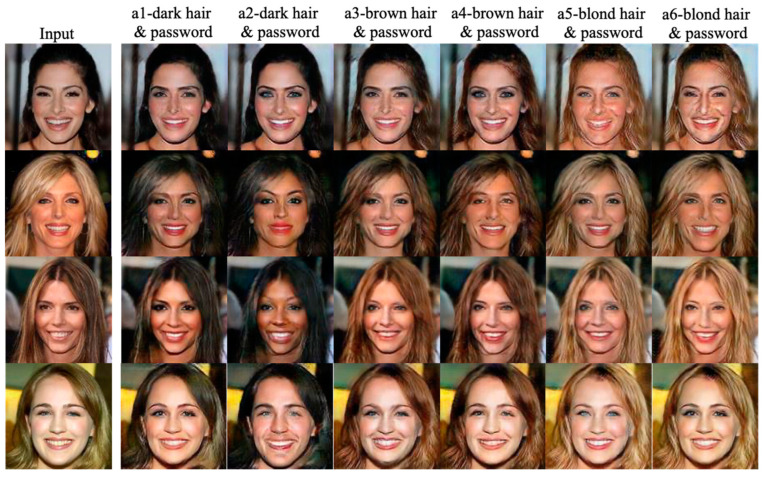
The generated agent faces using the password and multi-factor registration scheme with a fixed password and different hair colors (black, brown, and blond in this test).

In Figure 20, the first column shows the input images and the rest of the columns show the agent faces generated using the multi-factor combinations with a fixed password and different hair colors. The second and third columns’ hair colors are dark, while the hair colors of the fourth and the fifth columns are progressively becoming brown. Finally, the hair colors of the sixth and the seventh columns are near to blond.

**Figure 21:**
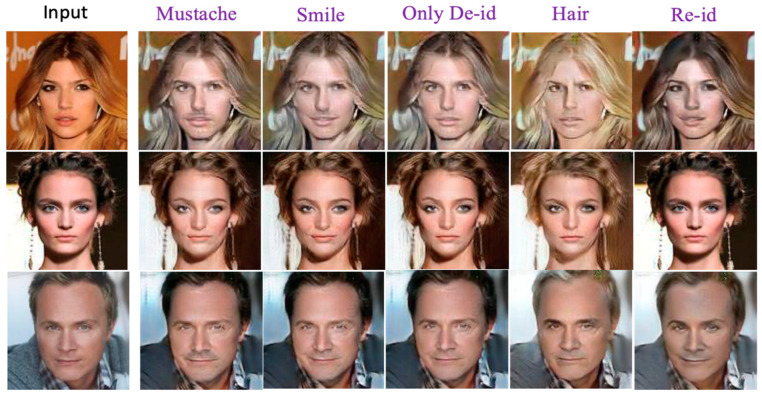
The results about the utility analysis with various combinations of face attributes and passwords for conducting the re-ID task.

Figure 21 illustrates the utility analysis results, including using a multi-factor combination with non-identity face attributes and passwords for generating de-ID images. For example, the second column shows the aggregating result of mustache and password, and the third column illustrates the results of smile plus password. The fourth column shows the generated de-ID images using various passwords only. The fifth column pitches the de-ID images using multi-factor combinations with identity-related face attributes (changing hair colors) and passwords. Notice that the last column presents the reconstructed images. Finally, leveraging the available facial recognition tools (as mentioned in Section 5.1) for evaluating our utility quality, Table 3 reports the distances between the anonymized images and the input images are rather far, especially when the multi-factor combination is involved.

**Figure 22:**
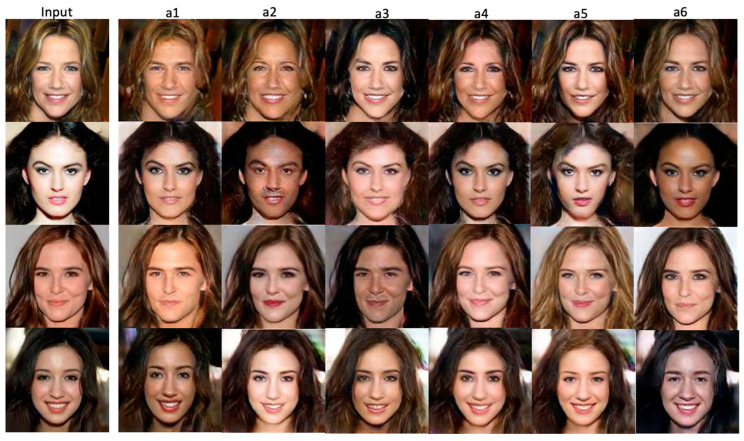
The impacts of similar passwords on a given anonymized image. The first column shows the input images and the second to seventh columns are the generated anonymized images with different passwords (from left to right, there is a 1-bit difference between the passwords used for input image a*_i_* and a*_i_*_+1_).

The following experiment examines the impacts of similar passwords on a given anonymized image. Under this testing situation, our system randomly selects some input images from the benchmark datasets and generates anonymized images by inputting different passwords with a 1-bit difference each. Figure 22 illustrates the outcomes of this experimental setting. The first column shows the input images, and the second to seventh columns are the generated anonymized images concerning different 1-bit difference passwords.

Using the face recognition tool [41], this test can be viewed as the objective counterpart associated with analyzing results, as presented in Figure 22. Table 4 lists the corresponding results. In the test, the default upper bound tolerance value is 0.6 [41], and, the smaller the value, the closer the faces are compared. As indicated in Table 4, the generated anonymized images are not so similar to each other if the password scheme is considered only. Even though two out of twenty-four values are smaller than 0.4, as indicated in Table 4, the overall performance is rather good (the successful recognition rate = overall average/0.6 = 0.499/0.6 > 83%). This good behavior comes from the fact that we dynamically generate sets of similar-feature-image groups concerning each labeled attribute in our original algorithm design. Thus, passwords have much less effect on quality of generated images than other facial features. In other words, we can treat the password as an additional feature for training the dataset.

**Figure 23:**
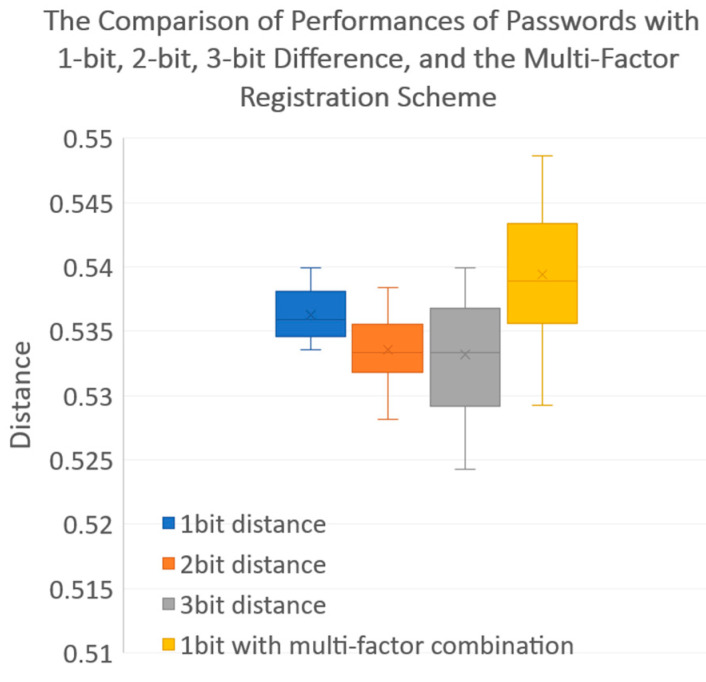
Comparison of performances (in terms of successful recognition rate) of passwords with 1-bit, 2-bit, and 3-bit differences and the proposed multi-factor registration scheme.

Last, let us discuss the complexity and effects of password length. Intuitively, we think the performance (in terms of the successful recognition rate or, equivalently, the degree of security level) of passwords with 2-bit or many-bit differentials would be better than passwords with a 1-bit differential, which means the resultant distance should be closer to the upper bound (0.6). Figure 23 shows that passwords’ complexity and bit length can only have a limited effect on degree of security, so verification through multi-factor combinations has higher priority in providing security guarantees.

## 6. Conclusions

We propose a new controllable and reversible Multi-factor Modifier (MfM) system to protect sensitive personal information. Unlike most related works, the proposed work anonymizes images and de-anonymizes images stably with a single neural network based on DosGAN and provides a security guarantee. Moreover, users can input the other face attributes as a multi-factor combination besides their passwords.

Justified by a series of experiments, the proposed reversible privacy protection system can precisely achieve facial anonymization and de-anonymization with the aid of the enhanced Multi-factor Modifier. According to the experiment, we can conclude that the LPIPS, FID, and SSIM values of MfM work have significantly better de-identification effects than the state-of-the-art methods through quantitative results. Furthermore, we also compare the proposed work with the anonymization performance of other de-ID-related works, including CIAGAN, k-Same-Net, and k-Same-Siamese-GAN. The performance of the proposed MfM is relatively good, and the synthesized agent face is also more realistic. This part is also in line with the inferences of our theoretical analysis through MI. In the face verification part, we compared the original images with the de-ID images and the re-ID images using state-of-the-art face recognition methods insightface, insightface_ir50 ms1m, and Facenet. The three networks showed 100% on the successful de-ID facial pictures. Under the de-anonymization situation, the MfM system’s outputs achieved a relatively positive gain in insightface (91.3%). The proposed MfM produces the best de-ID and re-ID results using the insightface model. We also report that the RMSE value is 0.353 and the accuracy value is 0.875.

On the other hand, we use qualitative evaluation results of MfM concerning utility-related experiments, including the effects of using the multi-factor registration scheme with face-identity-related attributes, style-related attributes, and a mixed scheme. Based on the above qualitative evaluation results, the designed MfM has very positive markings. Experiments have shown that specific features can be easily adjusted through latent space manipulation, and multi-factor security can be achieved by setting passwords. It is worth mentioning that, even if the designed password is only 1 bit different, the degree of difference between the generated proxy faces is substantial, which also echoes that we can maximize the dissimilarity anonymization images.

Moreover, we show that our system can successfully anonymize images in many cases, create photo-realistic faces satisfying the given conditions, and de-anonymize face images with the help of multi-factor attributes, as demonstrated by extensive experiments and comparisons with other competing methods as well. The superior performance of the proposed work results from including newly designed MI-based cost functions. Finally, information-theoretic-based mathematical derivations are presented in detail to enunciate our design insights and provide a better explanation to the readers.

Aside from MI, there are other helpful information-theoretic quantities, such as rate-distortion measures and channel capacity. How to incorporate those physically meaningful measures to polish further the effectiveness and efficiency of a de-ID and re-ID system is, of course, one of our future research directions.

## Figures and Tables

**Table 1:** The identity-related and style-related attributes involved in our testing case 2.

Attribute Classifications	Detail Attributes
ID-related attributes (labeled features)	Race; Age- {Old, Young}; Gender- {Male, Female}
Style-related attributes (labeled features)	Eyebrow {Arched_Eyebrows, Bushy_Eyebrows}; Eyeglasses; Bags_Under_Eyes; Big_Nose; Hair-Color {Black_Hair, Blond_Hair, Brown_Hair, Gray_Hair}; Beard {With_Mustache, No_Beard}; Mouth_Slightly_Open; Thick_Lips: Expression {Smiling; Expressionless}

**Table 2:** Perceptual quality comparisons: anonymization.

Method	LPIPS	FID	SSIM
k-Same-Siamese-GAN [16]	0.176	70.1	0.78
Password-conditional [19]	0.17	110	0.87
FaceBERT [20]	0.15	123	0.83
Yang et al. [21]	0.12	144	0.81
A^3^GAN [22]	0.29	93	0.87
Khorzooghi et al. [23]	0.288	101	0.86
Xue et al. [24]	0.16	127	0.83
CIAGAN [43]	0.281	108	0.85
Cao et al. [44]	0.29	43	0.93
Ours	0.35	28	0.95
Real Images	-	15.1	1

**Table 3:** The distances between the de-ID faces generated by the multi-factor combination scheme and their re-ID counterparts associated with the test shown in Figure 21.

Images from Figure 20	Mustache	Smile	Only de-ID	Hair	re-ID
Top Input Image	0.581	0.553	0.543	0.571	0.216
Middle Input Image	0.510	0.490	0.459	0.494	0.195
Bottom Input Image	0.504	0.499	0.453	0.522	0.234

**Table 4:** The objective distances between the anonymized images and the original inputs measured by using the well-performed face recognition tool presented in [41], associated with test given inputs from Figure 22.

Inputs from Figure 21	a1	a2	a3	a4	a5	a6
The 1st Input	0.554	0.517	0.560	0.532	0.453	0.485
The 2nd Input	0.510	0.512	0.551	0.427	0.389	0.575
The 3rd Input	0.557	0.387	0.555	0.524	0.498	0.459
The 4th Input	0.406	0.527	0.467	0.558	0.519	0.445
